# Supplementary material for: Intestinal interleukin-22 enhances GLP-1 production via the STAT3 pathway to improve glucose homeostasis during high-fat diet induced obesity in a study with male mice
Source: Nat Commun. 2026 Feb 21;17:3009. doi: 10.1038/s41467-026-69734-0 (PMC13035814; doi:10.1038/s41467-026-69734-0)
Supplement: Supplementary file 1 — Supplementary_information [file 41467_2026_69734_MOESM1_ESM.pdf]

Intestinal interleukin-22 enhances GLP-1 production via the STAT3 pathway to improve glucose homeostasis during high-fat diet induced obesity in a study with male mice

Chae-Won Kim[1,†], Jae-Hee Ahn[1,†], Bo Ra Lee[1,2,†], Hong Min Kim[3,†], Youngjoo Han[1], Jae-Hyeon Jeong[1], Jaewon Cho[1], Hyunjin Jeong[1], Dae-Joon Kim[1], Seong-Eun Kim[1], Jeon-Kyung Kim[4], Yu-Bin Lee[4], Su Min Kim[5], Hye Hyun Yoo[5], Eun Hye Lee[6], Su Ryeon Seo[6], Kyung Bong Ha[3], Eun Soo Lee[3], Mi-Na Kweon[7], Hong Pyo Kim[8], Sun-Young Chang[8], Choon Hee Chung[3]*, Hyun-Jeong Ko[1,9,10]*

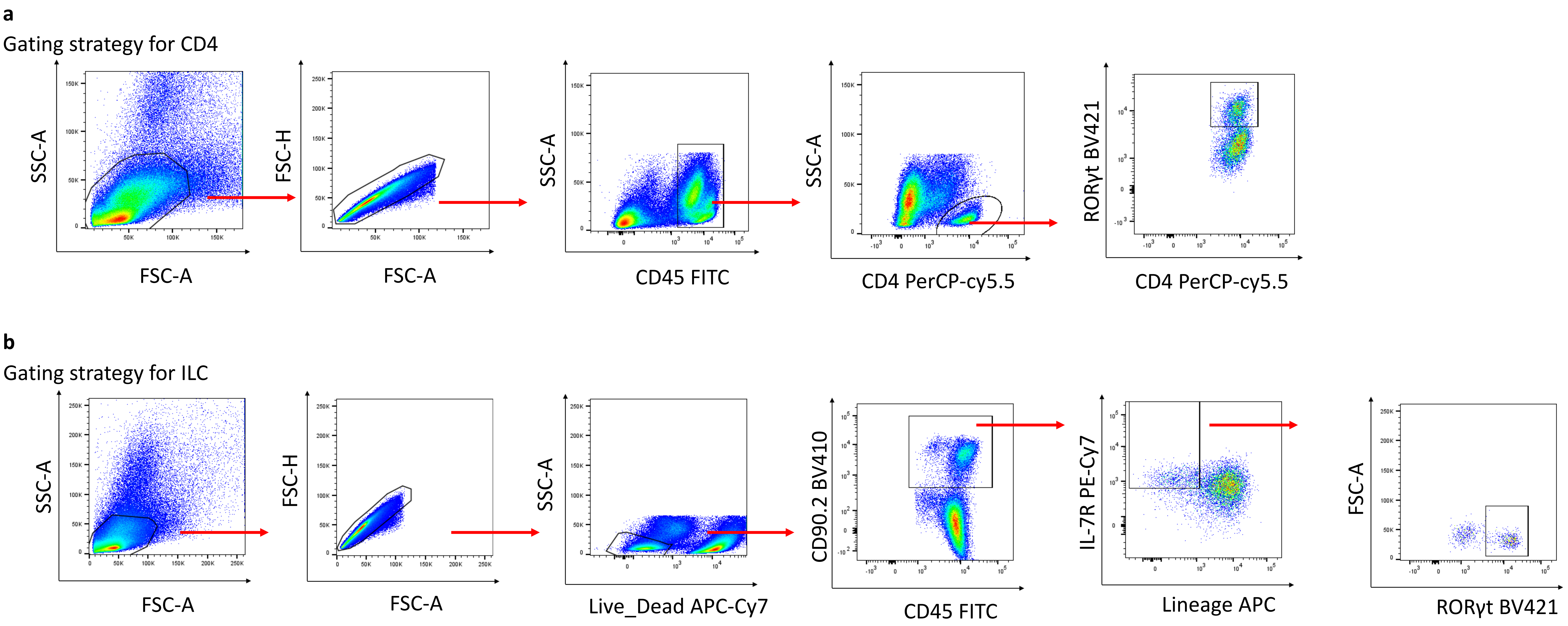

Supplementary figure 1

(**a**) Gating strategy for CD4+ T cells. Lymphocytes were first identified using an SSC-A versus forward FSC-A plot. Doublets were excluded using FSC-H versus FSC-A. CD45+ leukocytes were gated, followed by a selection of CD4+ T cells using CD4 PerCP-Cy5.5. The expression of RORγt BV421 within the CD4+ population was analyzed. (**b**) Gating strategy for ILCs. Lymphocytes were identified using SSC-A versus FSC-A. Doublets were excluded using FSC-H versus FSC-A. Dead cells were excluded using Live/Dead APC-Cy7 staining. CD45+ and CD90.2+ cells were gated to identify ILCs. Further gating was performed on IL-7Rα PE-Cy7+ and Lineage APC- cells. Finally, RORγt BV421 expression was analyzed to characterize specific ILC subsets.

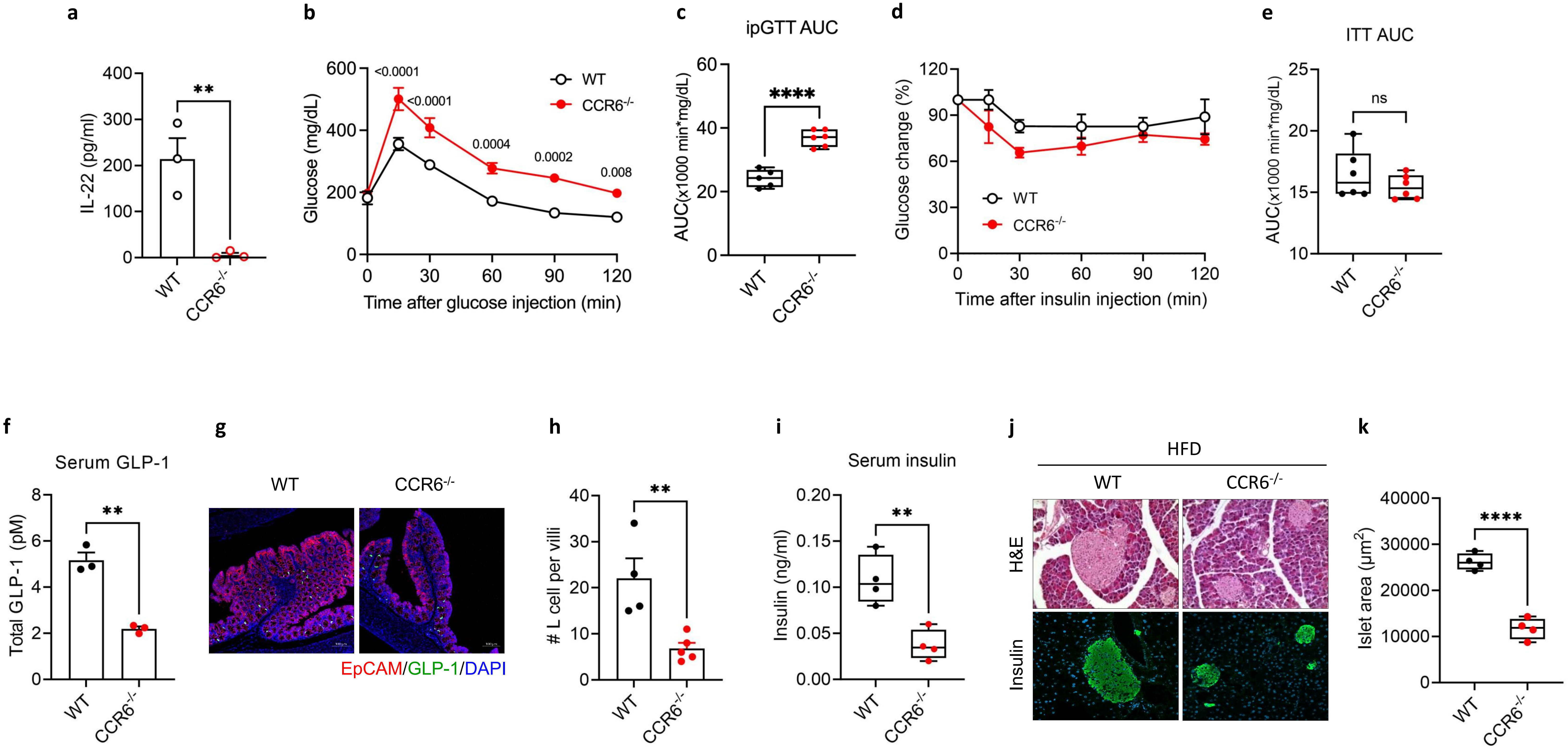

Supplementary figure 2

(**a**) The levels of intestinal IL-22 in WT (n = 3) and CCR6-/- mice (n = 3) ($P = 0.005$). (**b**) WT (n = 5) and CCR6$^{-/-}$ mice (n = 6) underwent intraperitoneal glucose (1 g/kg) tolerance tests (IPGTT) at 8 weeks. (**c**) The area under the curve (AUC) during IPGTT ($P < 0.0001$). (**d**) Insulin (1 U/kg) tolerance tests (ITT) in 6-h fasted mice at 10 weeks. (**e**) The AUC during ITT. (**f**) The serum levels of GLP-1 (WT: n = 3, CCR6$^{-/-}$: n = 3) ($P = 0.001$). (f) Immunofluorescence staining of the mouse intestines for EPCAM (red), GLP-1 (green), and nuclei (DAPI, blue). Original magnification 20×. (**h**) The number of L cells ($P = 0.007$). (**i**) Serum insulin levels in WT and CCR6$^{-/-}$ mice (WT: n = 4, CCR6$^{-/-}$: n = 4) ($P = 0.004$). (**j**) H&E staining images of the mouse pancreas, along with immunofluorescence staining images. Original magnification 20× (scale bar, 50 μm). (**k**) Quantification of pancreatic islet ($P < 0.0001$). Statistical significance was analyzed using an unpaired two-tailed Student's t-test (**a, c, f, h, i, k**) and 2way ANOVA (**b**). $P < 0.05$ (*), $P < 0.01$ (**), $P < 0.001$ (***), $P < 0.0001$ (****). Data are presented as mean ± SEM. Box plots show the median (center line), the 25th and 75th percentiles (box), and the minimum and maximum values (whiskers).

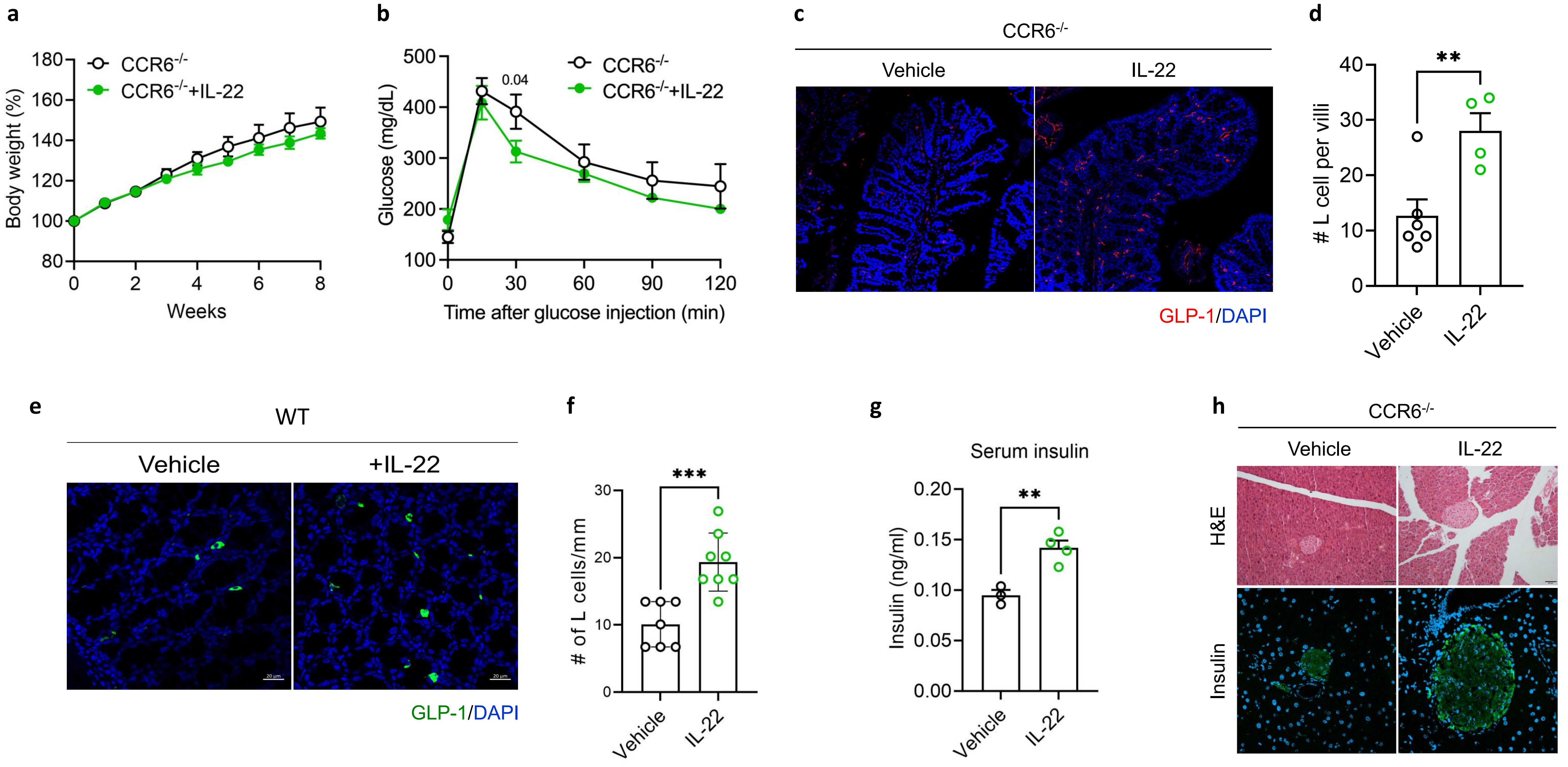

Supplementary figure 3

Long-term IL-22 treatment restores metabolic and enteroendocrine defects in HFD-fed CCR6$^{-/-}$ mice. CCR6$^{-/-}$ mice were fed an HFD and administered PBS or IL-22 (20 µg/kg, i.p.) weekly for 8 weeks. (**a**) Weekly body weight (n = 5 per group). (**b**) IPGTT after a 16-h fast at week 8. (**c**) GLP-1 (red) and DAPI staining in intestines of HFD-fed CCR6$^{-/-}$ mice. (**d**) Quantification of GLP-1^{+} L cells in c ($P = 0.009$). (**e**) GLP-1 (green) and DAPI staining in HFD-fed WT mice treated with IL-22 (n = 7) or vehicle (n = 8). (**f**) Quantification of GLP-1^{+} L cells in e ($P = 0.0005$). (**g**) Serum insulin levels in CCR6$^{-/-}$ mice with or without IL-22 (WT: n = 3; CCR6$^{-/-}$: n = 4) ($P = 0.004$). (**h**) H&E staining and insulin immunofluorescence of pancreas (20×; scale bar, 50 µm). Data are mean ± SEM. Statistical analyses were performed by two-way ANOVA (b) or unpaired two-tailed Student's t-test (d, f, g). $P < 0.05$ (*); $P < 0.01$ (**), $P < 0.001$ (***).

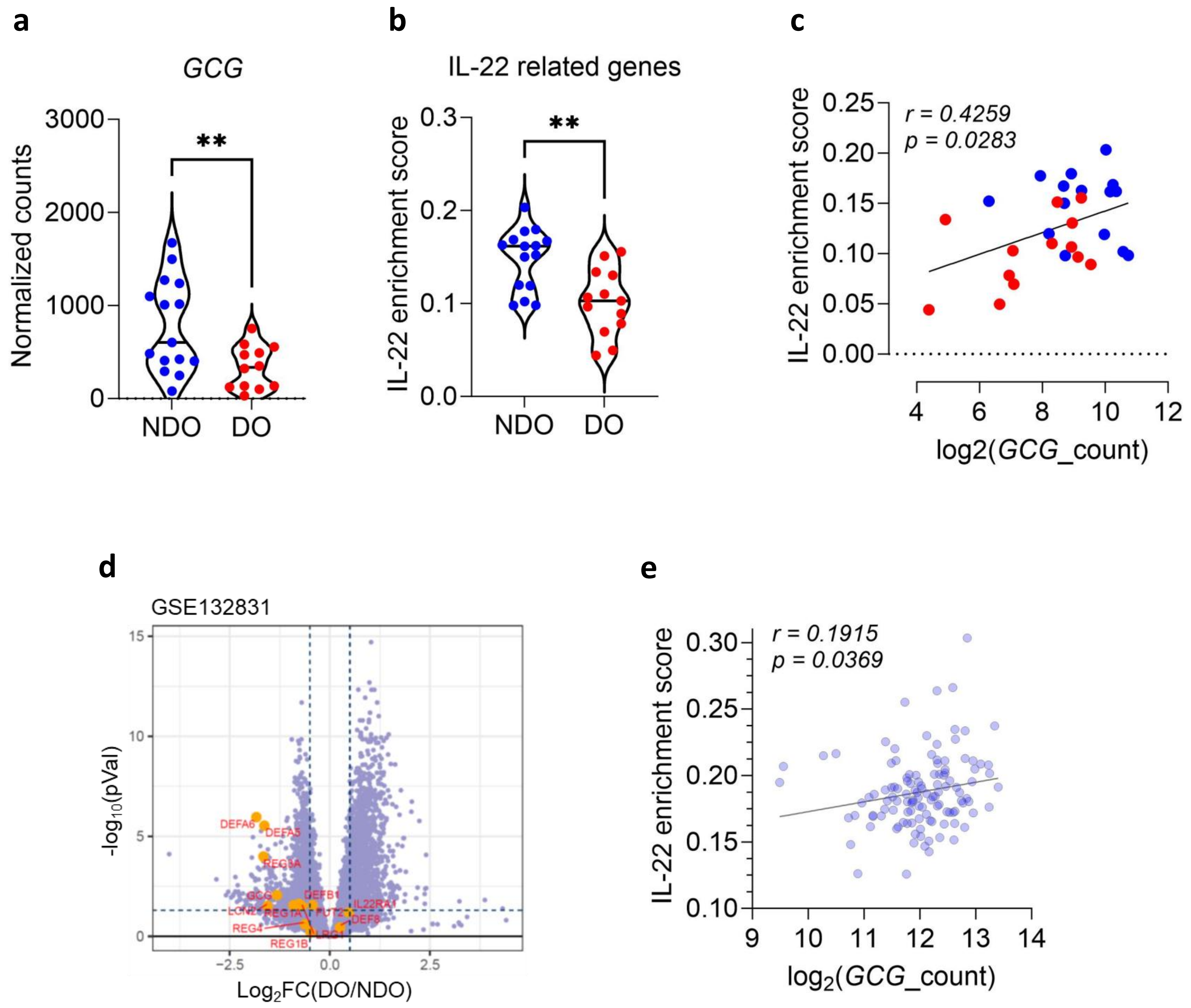

Supplementary figure 4

Comparison of the (**a**) GCG RNA count (*P* = 0.004) and (**b**) IL-22–responsive transcript enrichment scores (*P* = 0.001) derived from ileal biopsy RNA-seq profiles of non-diabetic individuals and obese patients with diabetes (GSE132831). The "IL-22–related gene set" included *REG3B, REG3G, SOCS3, FUT2, MUC1, SAA3, S100A8, LRG1, S100A9, SAA1, SAA2, IL10RB, RORC, AHR,* and. *STAT3*(**c**) A scattered plot of ileal GCG RNA counts and IL-22-responsive transcript enrichment scores obtained from ileal biopsies of non-diabetic patients and obese patients with diabetes, as determined based on the RNAseq dataset GSE132831. (**d**) A volcano plot of differential gene expression (fold change) and p-values, as determined based on the RNAseq dataset GSE132831. IL-22-associate genes are indicated by yellow points. (**e**) A scattered plot of ileal GCG RNA counts and IL-22-responsive transcript enrichment scores obtained from ileal biopsies of healthy controls (RNAseq dataset GSE193677). Statistical significance was analyzed using an unpaired two-tailed Student's t-test (**a**, **b**). *P* < 0.01 (**).

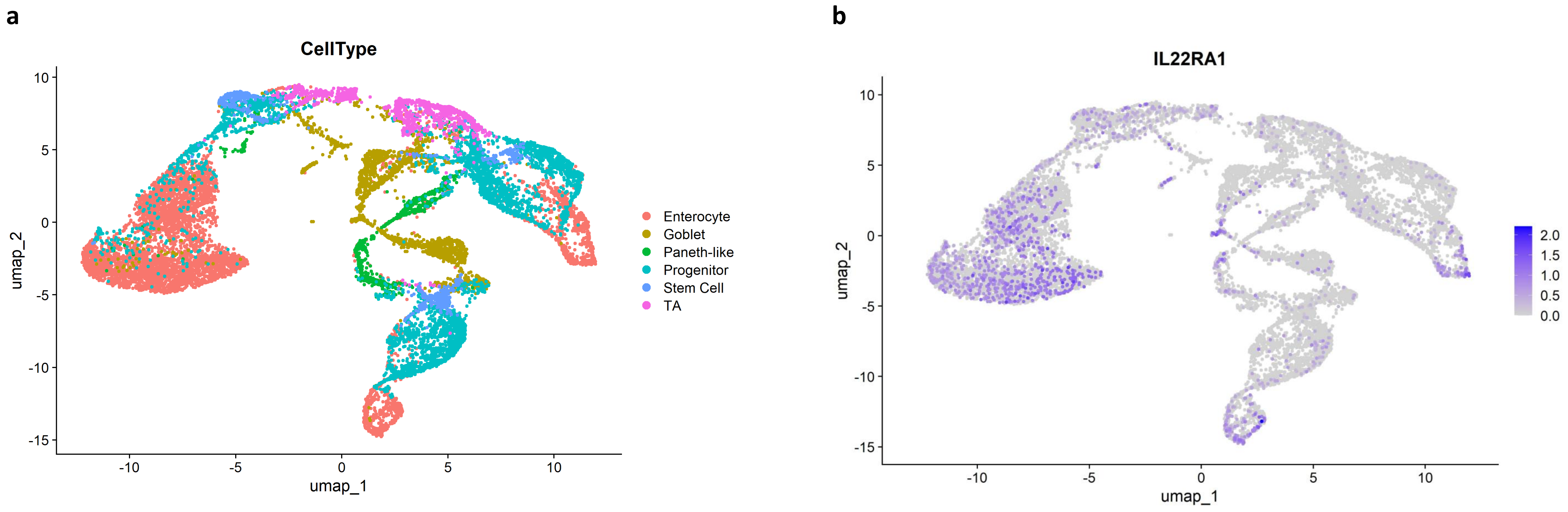

Supplementary figure 5

Single-cell RNA sequencing data from the GSE125970 dataset. (**a**) The UMAP plot represents individual clusters of identified intestinal epithelial cells. (**b**) The right panel shows the expression levels of a specific gene within the same cell population. Cells are color-coded based on expression levels (CPM - Counts Per Million), with the gradient ranging from light blue (low expression) to dark blue (high expression).

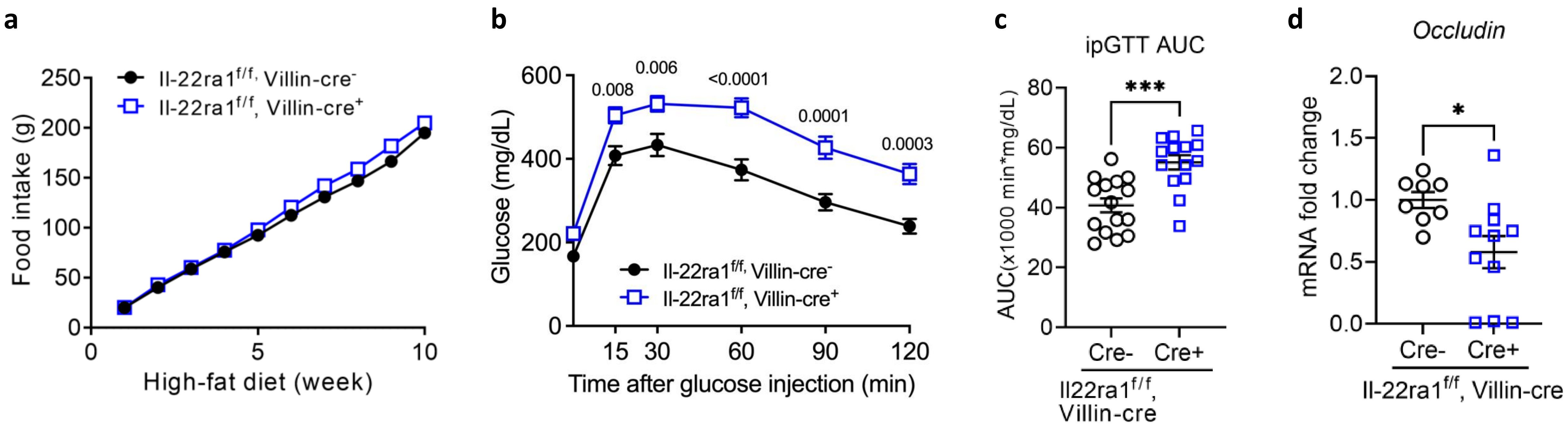

Supplementary figure 6

(**a**) Food intake of HFD-fed IL-22RA1$^{(f/f)}$ and IL-22RA1$^{Vil\ KO}$ mice. (**b**) Intraperitoneal glucose (1 g/kg) tolerance test (IPGTT) in 16 h-fasted mice at 8 weeks. (**c**) The area under the curve (AUC) during ipGTT (P = 0.0002). mRNA levels of (**d**) *Occludin* expression in the small intestines of IL-22RA1$^{(f/f)}$ (n = 8) and IL-22RA1$^{Vil\ KO}$ (n = 11) mice (P = 0.01). Statistical significance was analyzed using 2way ANOVA (**b**) and unpaired two-tailed Student's t-test (**c**, **d**). $P < 0.05$ (*), $P < 0.01$ (**), $P < 0.001$ (***). Data are presented as mean ± SEM.

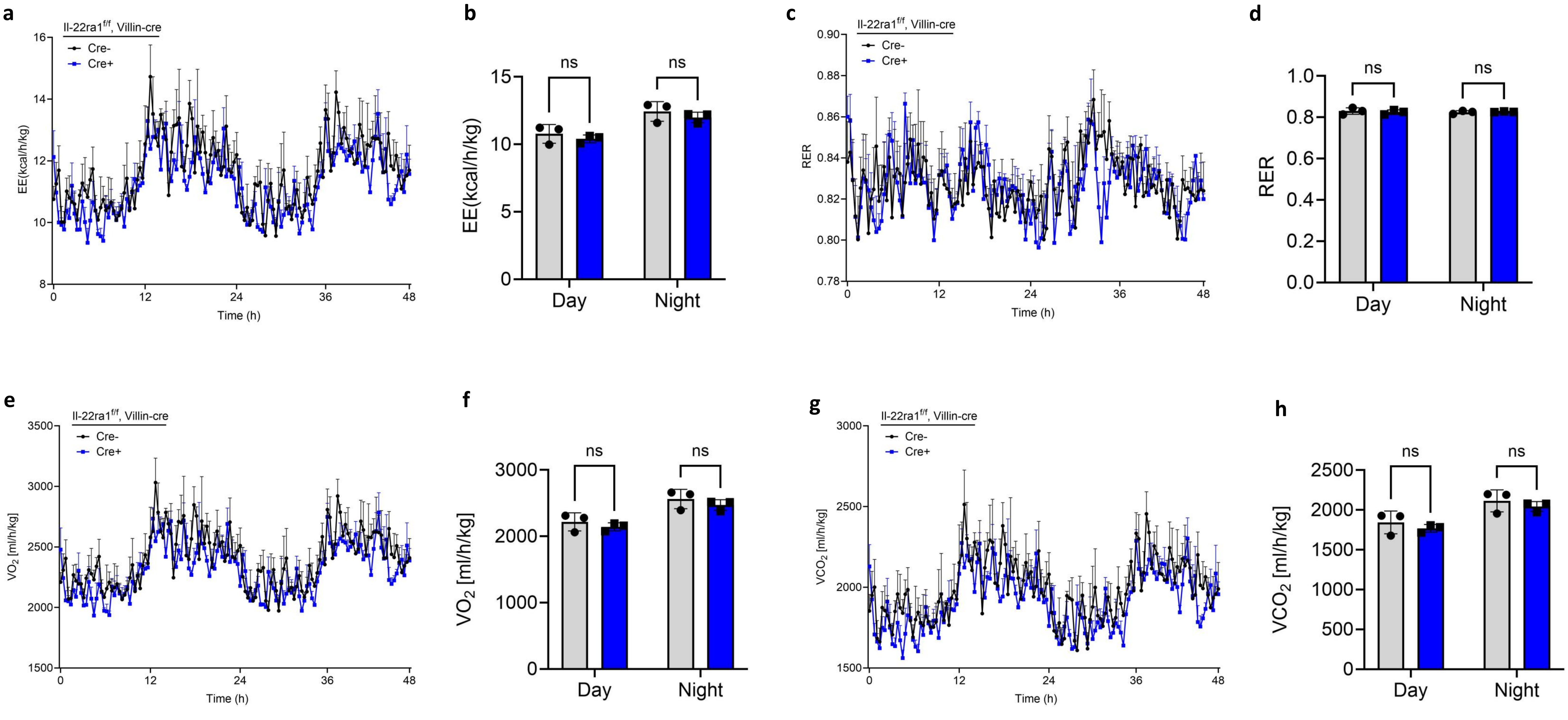

Supplementary figure 7

HFD-fed IL-22RA1$^{(f/f)}$ and IL-22RA1$^{Vil\ KO}$ mice (individually housed, n = 3/group) were housed in PhenoMaster metabolic cages (TSE Systems, Germany) at 23–24°C, with free access to food and water. After 24 h of acclimation, metabolic parameters were recorded continuously for 48 h under a 12 h light/12 h dark cycle. (**a**) Energy expenditure over 48 h (kcal/h).(**b**) Mean EE during light and dark phases. (**c**) Respiratory exchange ratio (RER) over 48 h. (**d**) Mean RER during light and dark phases.(e) Oxygen consumption (VO_2) over 48 h. (**f**) Mean VO_2 during light and dark phases. (**g**) Carbon dioxide production (VCO_2) over 48 h. (**h**) Mean VCO_2 during light and dark phases. Statistical significance was analyzed using an unpaired two-tailed Student's t-test. Data are presented as mean ± SEM.

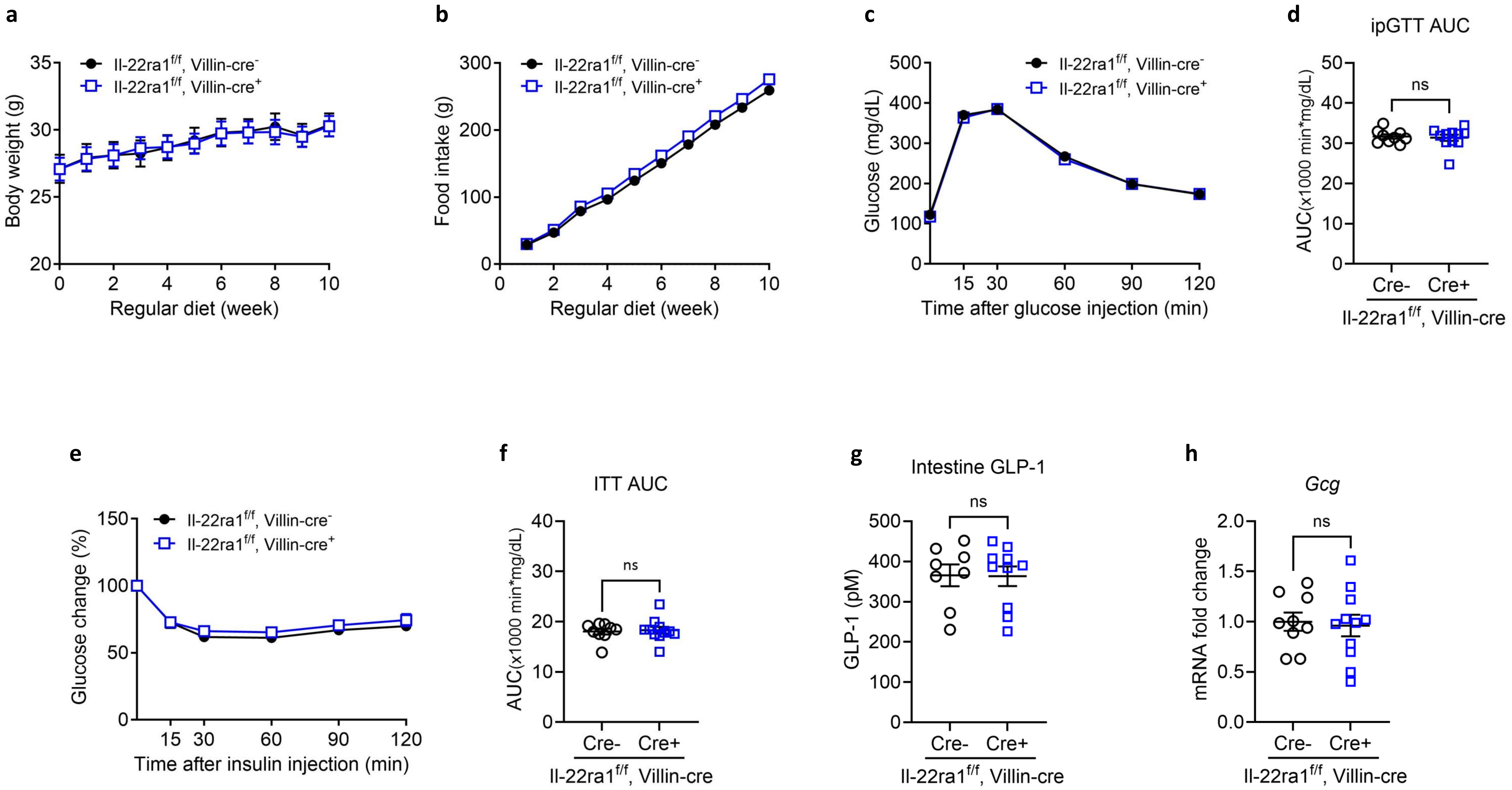

Supplementary figure 8

IL-22RA1$^{(f/f)}$ (n = 9) and IL-22RA1$^{Vil\ KO}$ (n = 11) mice were fed an RD for 12 weeks. (**a**) Weekly bodyweight changes of IL-22RA1$^{(f/f)}$ and IL-22RA1$^{Vil\ KO}$ mice. (**b**) Food intake per mouse. (**c**) IPGTT results in 16-h fasted mice at 8 weeks. (**d**) The AUC during ipGTT. (**e**) ITT results in 6-h fasted mice at 10 weeks. (**f**) The AUC during ITT. (**g**) The levels of GLP-1 extracted from the mouse intestinal supernatant. mRNA levels of (**h**) *Gcg* in the mouse small intestine. Statistical significance was analyzed using an unpaired two-tailed Student's t-test. ns, not significant. Data are presented as mean ± SEM. Representative data are shown from two independent experiments

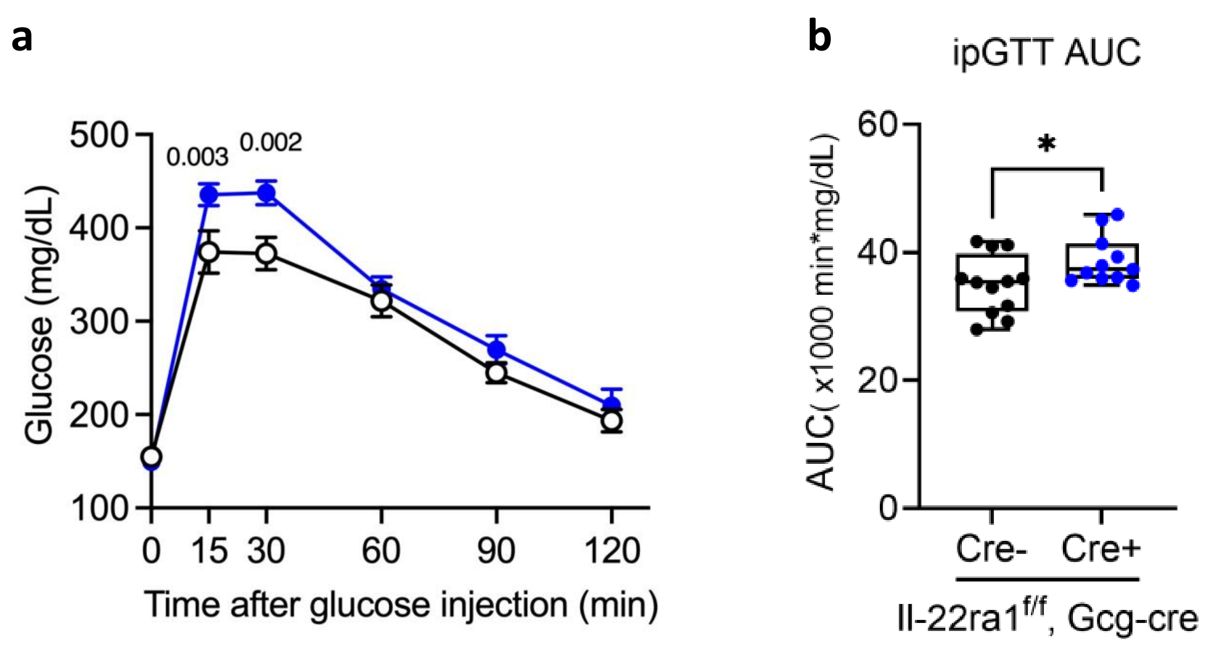

Supplementary figure 9

(**a**) IPGTT in 16-h-fasted mice at 8 weeks. (**b**) The AUC during ipGTT ($P = 0.04$). Statistical significance was analyzed using (**a**) 2way ANOVA and (**b**) unpaired two-tailed Student's t-test. $P < 0.05$ (*), $P < 0.01$ (**). Data are presented as mean ± SEM. Box plots show the median (center line), the 25th and 75th percentiles (box), and the minimum and maximum values (whiskers).

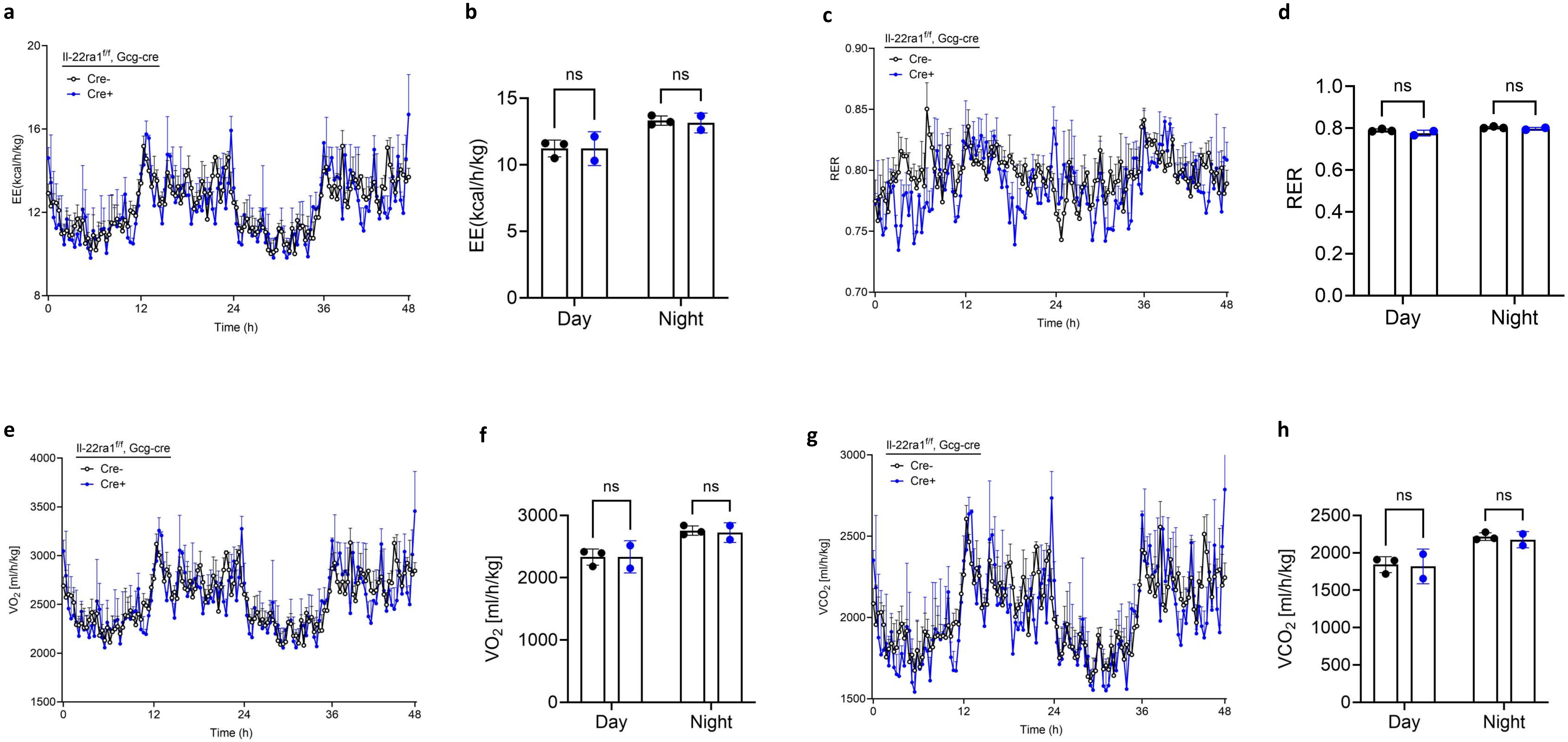

Supplementary figure 10

HFD-fed IL-22RA1$^{(f/f)}$ and IL-22RA1$^{Gcg\ KO}$ mice (individually housed, n = 3/group) were housed in PhenoMaster metabolic cages (TSE Systems, Germany) at 23–24°C, with free access to food and water. After 24 h of acclimation, metabolic parameters were recorded continuously for 48 h under a 12 h light/12 h dark cycle. (**a**) Energy expenditure over 48 h (kcal/h).(**b**) Mean EE during light and dark phases. (**c**) Respiratory exchange ratio (RER) over 48 h. (**d**) Mean RER during light and dark phases.(e) Oxygen consumption (VO_2) over 48 h. (**f**) Mean VO_2 during light and dark phases. (**g**) Carbon dioxide production (VCO_2) over 48 h. (**h**) Mean VCO_2 during light and dark phases. Statistical significance was analyzed using an unpaired two-tailed Student's t-test. ns, not significant. Data are presented as mean ± SEM.

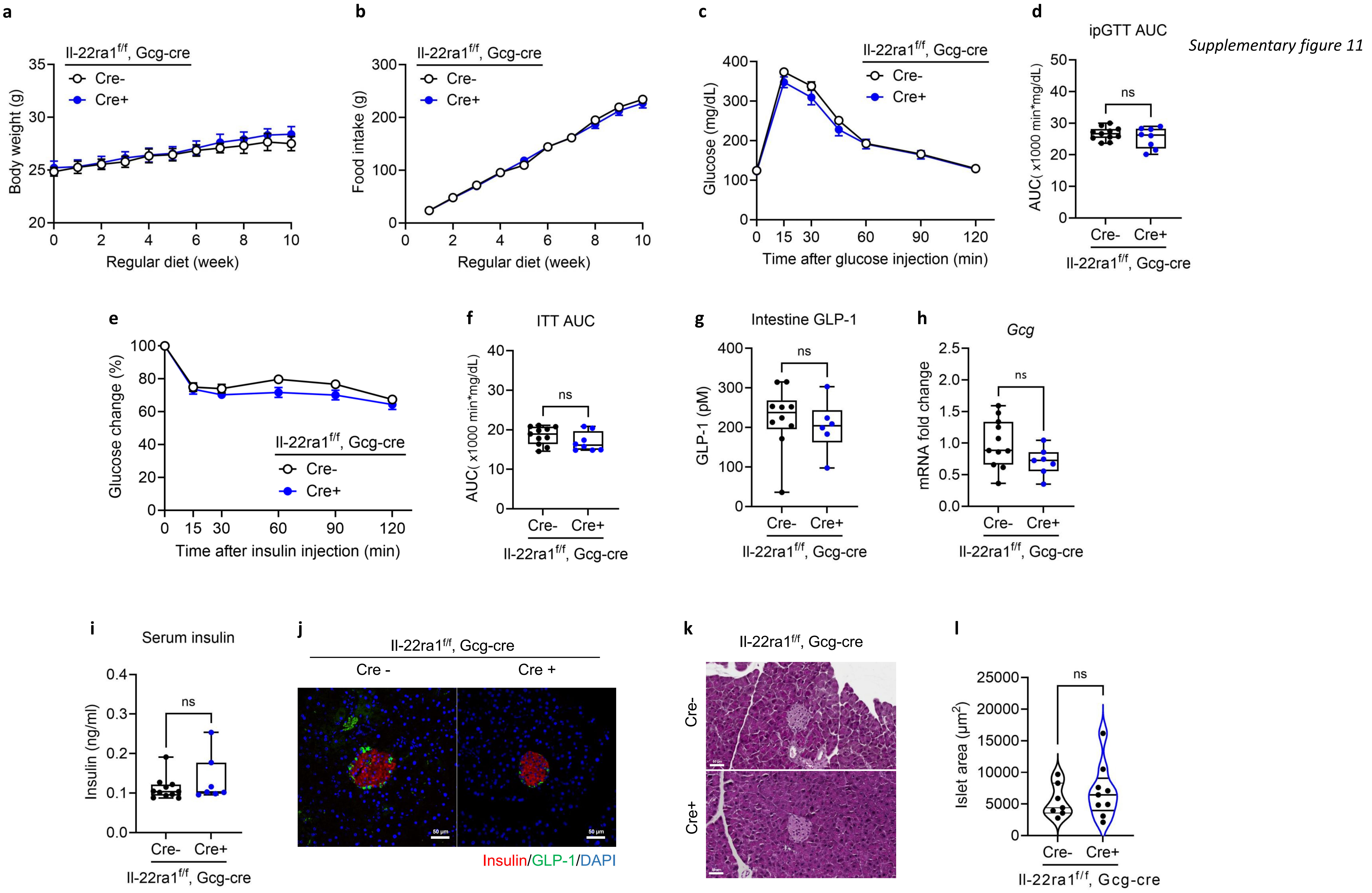

Supplementary figure 11

IL-22RA1$^{(f/f)}$ and IL-22RA1$^{Gcg\ KO}$ mice were fed an RD for 12 weeks. (**a**) Weekly bodyweight changes of IL-22RA1$^{(f/f)}$ and IL-22RA1$^{Gcg\ KO}$ mice. (**b**) Food intake per mouse. (**c**) IPGTT in 16-h fasted mice at 8 weeks. (**d**) The AUC during ipGTT. (**e**) ITT in 6-h fasted mice at 10 weeks. (**f**) The AUC during ITT. (**g**) The levels of GLP-1 extracted from the mouse intestinal supernatant. mRNA levels of (**h**) *Gcg* in the small intestines. (**i**) Serum insulin levels in IL-22RA1$^{(f/f)}$ and IL-22RA1$^{Gcg\ KO}$ mice. (**j**) Immunofluorescence staining image for insulin (red), GLP-1 (green), and nuclei (DAPI, blue) from mouse pancreas. Original magnification 20× (scale bar, 50 μm). (**k**) H&E staining of the mouse pancreas. Original magnification 20× (scale bar, 50 μm). (**l**) Quantification of the pancreatic islet. n = 5 (IL-22RA1$^{(f/f)}$), n = 5 (IL-22RA1$^{Gcg\ KO}$) for (**a**,**b**); n = 11 (IL-22RA1$^{(f/f)}$), n = 7 (IL-22RA1$^{Gcg\ KO}$) for (**c-l**). Statistical significance was analyzed using an unpaired two-tailed Student's t-test. ns, not significant. Data are presented as mean ± SEM. Box plots show the median (center line), the 25th and 75th percentiles (box), and the minimum and maximum values (whiskers). Representative data are shown from two independent experiments.

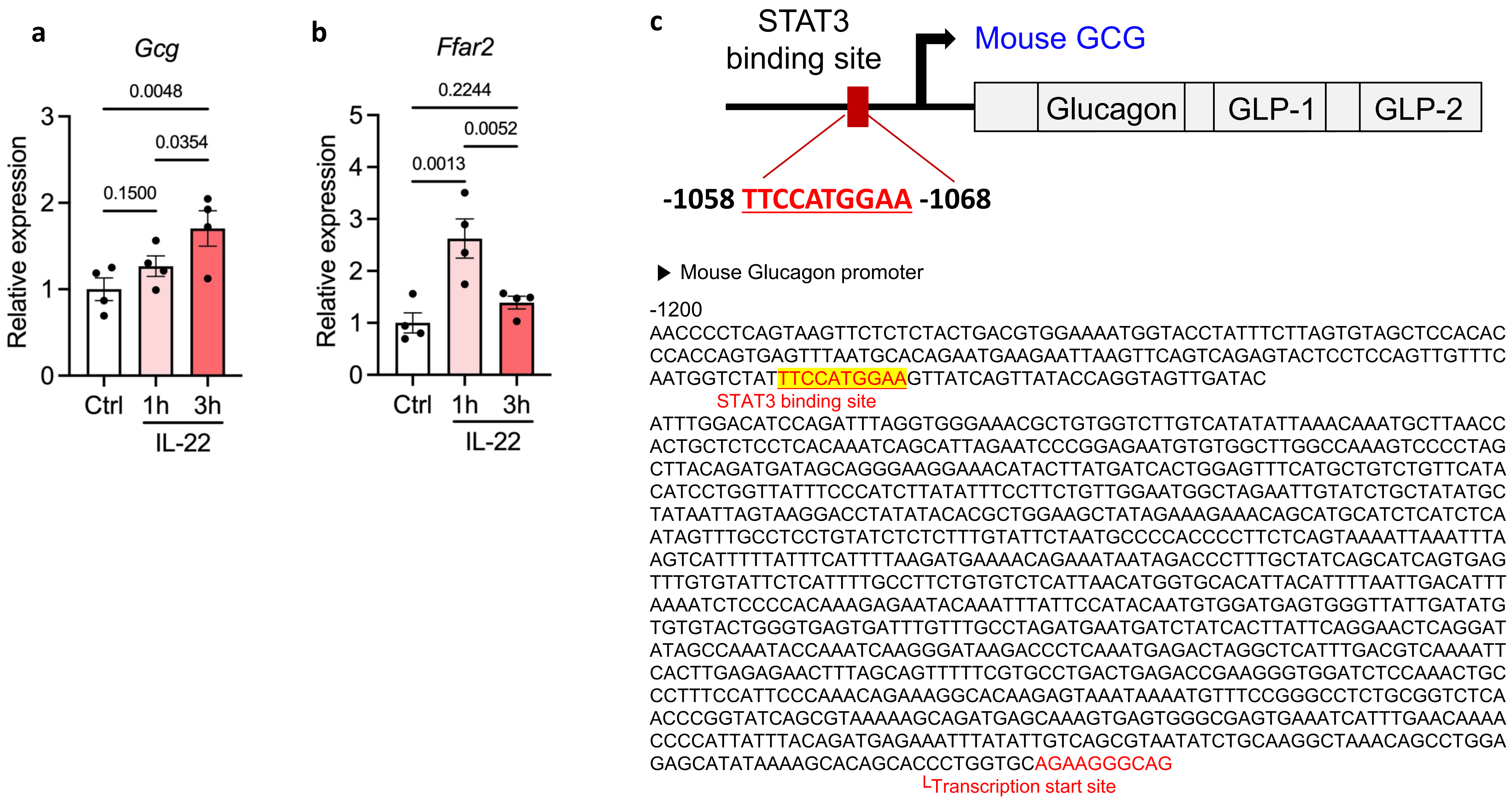

Supplementary figure 12

mRNA levels of (**a**) *Gcg* and (**b**) *Ffar2* expression were determined in STC-1 cells treated with IL-22 (10 ng/mL). (**c**) The structure and STAT3 binding site of the mouse glucagon (*GCG*) gene. A specific sequence, TTCCATGGAA, is identified as a STAT3 binding site and is highlighted in yellow and indicated in red text. The position of the STAT3 binding site relative to the transcription start site indicated by the AGAAGGGCAG sequence in red. Statistical significance was analyzed using Ordinary one-way ANOVA. $P < 0.05$ (*), $P < 0.01$ (**). ns, not significant. Data are presented as mean ± SEM.

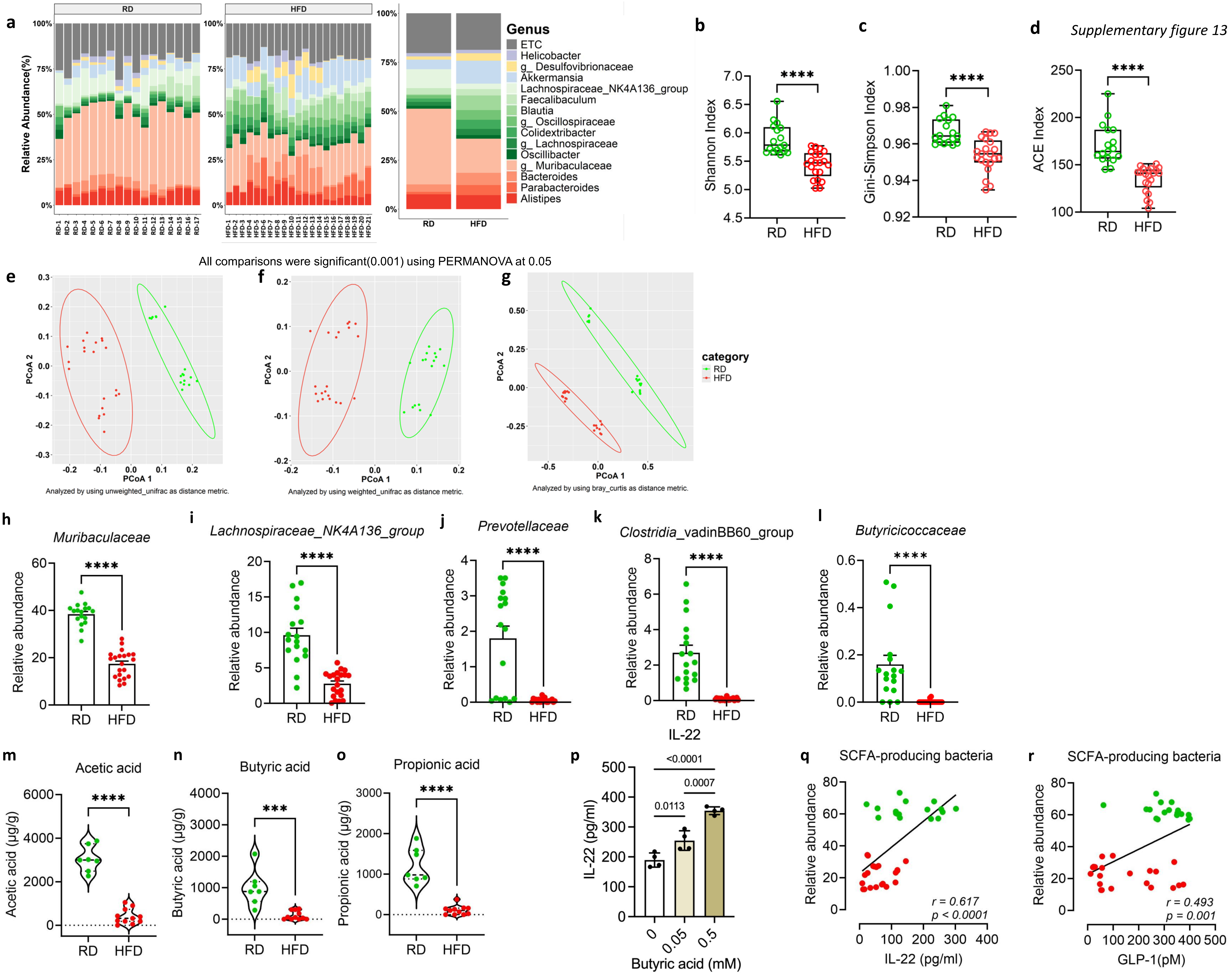

Supplementary figure 13

Supplementary figure 13

(**a**) Composition of the fecal microbiota in RD - or HFD - fed mice, as determined based on relative abundance at the genus level. A boxplot of Alpha diversity comparison of RD- or HFD-fed mice. Alpha diversity is represented by the (**b**) Shannon (P <0.0001), (**c**) Gini-Simpson (P <0.0001), and (**d**) ACE indices (P <0.0001). (**c**) Principal coordinate analysis plots comparing beta diversity between samples from RD- and HFD-fed mice. Each plot was generated using a different distance metric: (**e**) unweighted UniFrac, (**f**) weighted UniFrac, and (**g**) Bray–Curtis, respectively, to assess differences in microbial community composition. Comparison of the relative abundance of SCFA-producing bacteria in RD- or HFD-fed mice. (**h**) Muribaculaceae (P <0.0001) (**i**) Lachnospiraceae_NK4A136_group (P <0.0001) (**j**) Prevotellaceae (P <0.0001) (**k**) Clostridia_vadinBB60_group (P <0.0001) (**l**) Butyricicoccaceae (P <0.0001). Concentration of SCFAs in fecal samples from RD - or HFD - fed mice. (**m**) Acetic acid (P <0.0001) (**n**) Butyric acid (P = 0.0001) (**o**) Propionic acid (P <0.0001). (**p**) Concentration of IL-22 extracted from the supernatant of mouse LP cells treated with butyric acid and P/I (PMA/ionomycin) stimulation for 16 h. A graph showing the correlation between the relative abundance of SCFA-producing bacteria and (**q**) intestinal IL-22 and (**r**) GLP-1 levels in RD- or HFD-fed mice. n = 17 (RD), n = 21 (HFD) for (**a**–**o**, **q, r**); n = 7 (RD), n = 11 (HFD) for (**m-o**). Statistical significance was analyzed using an unpaired two-tailed Student's t-test (**b-d, h-o**) and Ordinary one-way ANOVA (**p**). P < 0.05 (*), P < 0.001 (***), P < 0.0001 (****). Data are presented as mean ± SEM. Box plots show the median (center line), the 25th and 75th percentiles (box), and the minimum and maximum values (whiskers). Representative data are shown from two independent experiments.

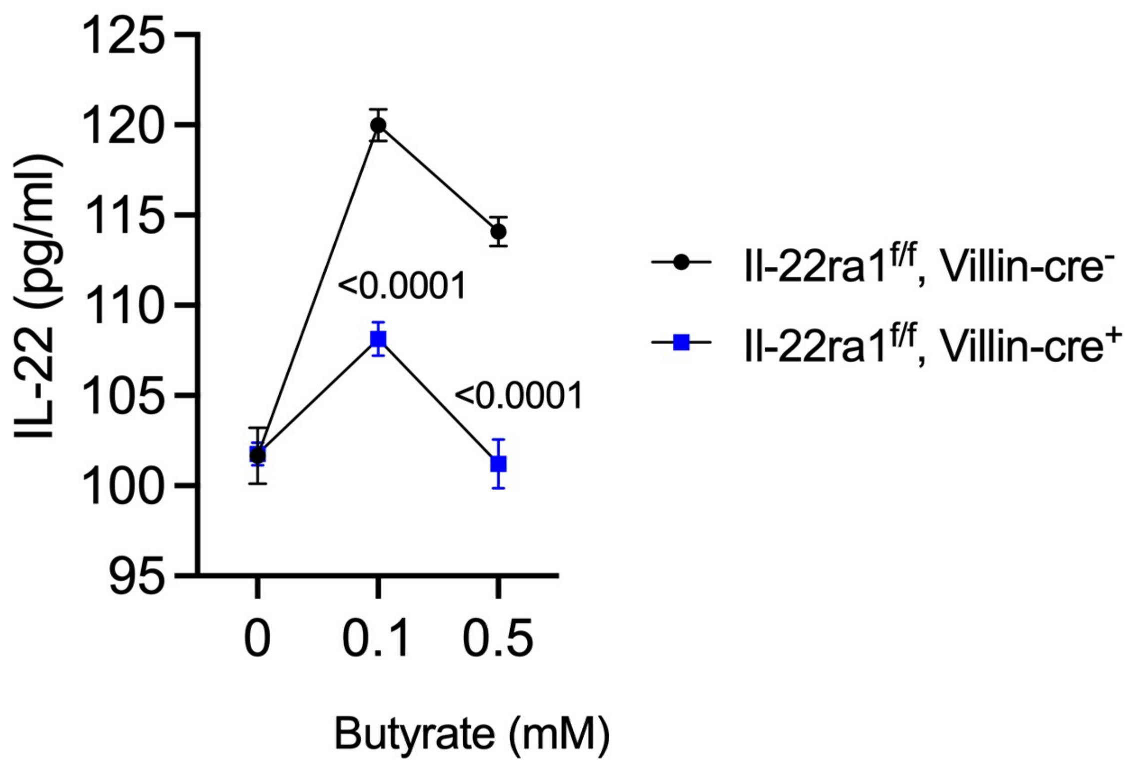

Supplementary figure 14

IL-22 concentrations measured in the supernatant of LP cells isolated from IL-22RA1$^{(f/f)}$ and IL-22RA1$^{Gcg\ KO}$ mice after 16 h of stimulation with butyrate (0, 0.1, or 0.5 mM). Statistical significance was analyzed using 2way ANOVA. $P < 0.0001$ (****). Data are presented as mean ± SEM.

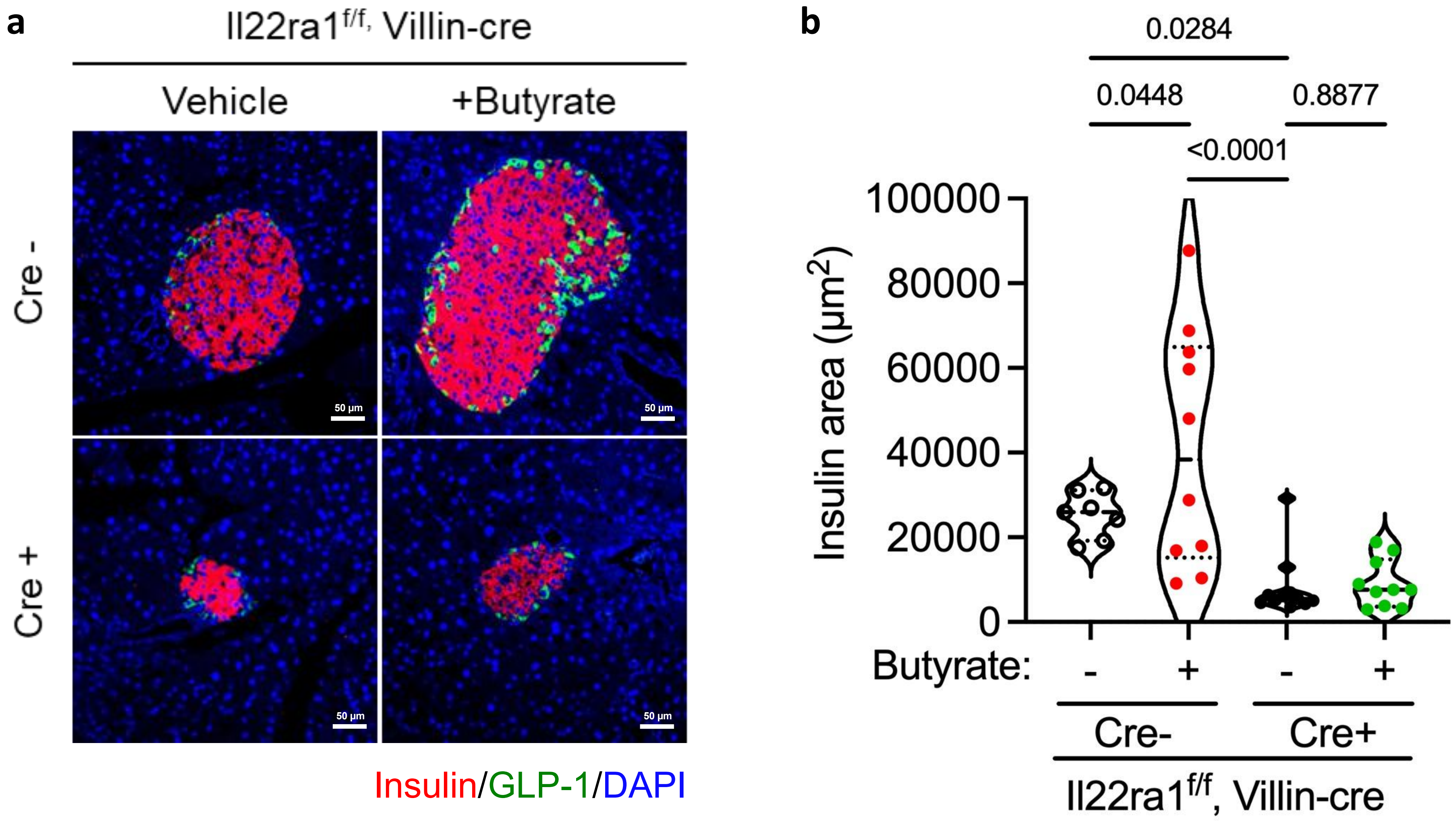

Supplementary figure 15

(**a**) Immunofluorescence staining image for insulin (red), GLP-1 (green), and nuclei (DAPI, blue) from mouse pancreas. Original magnification 20× (scale bar, 50 μm). (**b**) Quantification of the insulin-positive area. Statistical significance was analyzed using Ordinary one-way ANOVA. $P < 0.05$ (*), $P < 0.0001$ (****).ns, not significant. Data are presented as mean ± SEM.

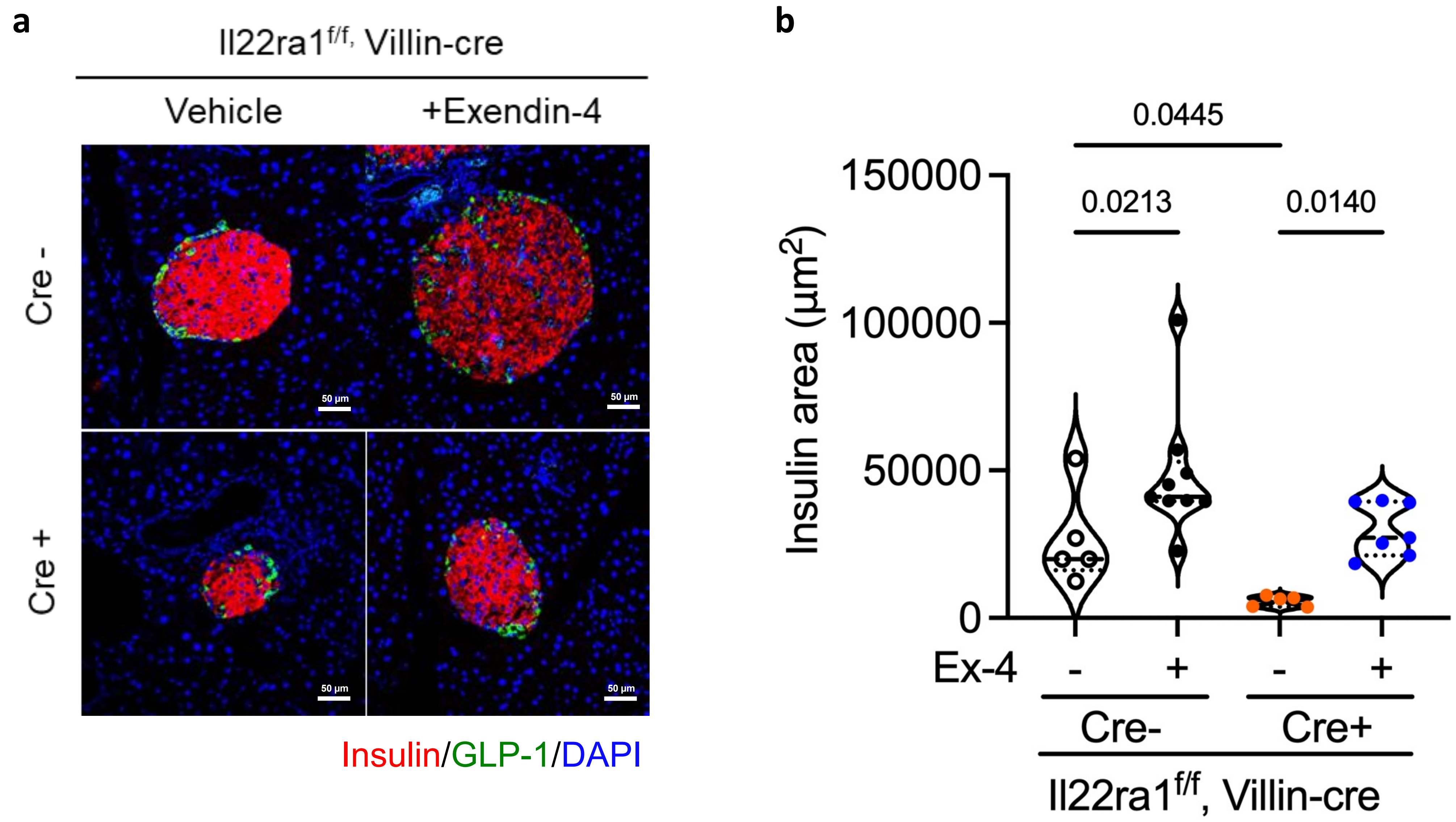

Supplementary figure 16

(**a**) Immunofluorescence staining image for insulin (red), GLP-1 (green), and nuclei (DAPI, blue) from mouse pancreas. Original magnification 20× (scale bar, 50 μm). (**b**) Quantification of the insulin-positive area. Statistical significance was analyzed using Ordinary one-way ANOVA. $P < 0.05$ (*). Data are presented as mean ± SEM.
