# Supplementary material for: Intestinal interleukin-22 enhances GLP-1 production via the STAT3 pathway to improve glucose homeostasis during high-fat diet induced obesity in a study with male mice
Source: Nat Commun. 2026 Feb 21;17:3009. doi: 10.1038/s41467-026-69734-0 (PMC13035814; doi:10.1038/s41467-026-69734-0)
Supplement: Supplementary file 2 — Reporting Summary [file 41467_2026_69734_MOESM2_ESM.pdf]

nature portfolio

Corresponding author(s): Hyun-Jeong Ko

Last updated by author(s): Dec 28, 2025

Reporting Summary

Nature Portfolio wishes to improve the reproducibility of the work that we publish. This form provides structure for consistency and transparency in reporting. For further information on Nature Portfolio policies, see our Editorial Policies and the Editorial Policy Checklist.

Statistics

For all statistical analyses, confirm that the following items are present in the figure legend, table legend, main text, or Methods section.

n/a	Confirmed	
☐	☒	The exact sample size (n) for each experimental group/condition, given as a discrete number and unit of measurement
☐	☒	A statement on whether measurements were taken from distinct samples or whether the same sample was measured repeatedly
☐	☒	The statistical test(s) used AND whether they are one- or two-sided<br>*Only common tests should be described solely by name; describe more complex techniques in the Methods section.*
☐	☒	A description of all covariates tested
☐	☒	A description of any assumptions or corrections, such as tests of normality and adjustment for multiple comparisons
☐	☒	A full description of the statistical parameters including central tendency (e.g. means) or other basic estimates (e.g. regression coefficient) AND variation (e.g. standard deviation) or associated estimates of uncertainty (e.g. confidence intervals)
☐	☒	For null hypothesis testing, the test statistic (e.g. F, t, r) with confidence intervals, effect sizes, degrees of freedom and P value noted<br>*Give P values as exact values whenever suitable.*
☒	☐	For Bayesian analysis, information on the choice of priors and Markov chain Monte Carlo settings
☒	☐	For hierarchical and complex designs, identification of the appropriate level for tests and full reporting of outcomes
☒	☐	Estimates of effect sizes (e.g. Cohen's d, Pearson's r), indicating how they were calculated

Our web collection on statistics for biologists contains articles on many of the points above.

Software and code

Policy information about availability of computer code

Data collection	Data were collected using BD FACSuite software (BD Biosciences) for flow cytometry acquisition. Illumina iSeq Control Software was used for 16S sequencing runs.
Data analysis	Data were analyzed using FlowJo (v10.10.0), GraphPad Prism (v10), and R (v4.4.1).16S rDNA amplicon sequencing data were processed using QIIME2 (version 2023.05) and the SILVA 138 database. GC–MS data acquisition and analysis were performed using MassHunter software (Agilent Technologies).

For manuscripts utilizing custom algorithms or software that are central to the research but not yet described in published literature, software must be made available to editors and reviewers. We strongly encourage code deposition in a community repository (e.g. GitHub). See the Nature Portfolio guidelines for submitting code & software for further information.

Data

Policy information about availability of data

All manuscripts must include a data availability statement. This statement should provide the following information, where applicable:

- Accession codes, unique identifiers, or web links for publicly available datasets
- A description of any restrictions on data availability
- For clinical datasets or third party data, please ensure that the statement adheres to our policy

All data supporting the findings of this study are included in the article and its Supplementary Information files. Raw 16S rDNA sequencing dat.a have been

deposited in the NCBI Sequence Read Archive (SRA) under accession number PRJNA1392864.

Research involving human participants, their data, or biological material

Policy information about studies with human participants or human data. See also policy information about sex, gender (identity/presentation), and sexual orientation and race, ethnicity and racism.

Reporting on sex and gender	No human participants, human tissues, or personally identifiable human data were used in this study.
Reporting on race, ethnicity, or other socially relevant groupings	No human participants, human tissues, or personally identifiable human data were used in this study.
Population characteristics	No human participants, human tissues, or personally identifiable human data were used in this study.
Recruitment	No human participants, human tissues, or personally identifiable human data were used in this study.
Ethics oversight	No human participants, human tissues, or personally identifiable human data were used in this study.

Note that full information on the approval of the study protocol must also be provided in the manuscript.

Field-specific reporting

Please select the one below that is the best fit for your research. If you are not sure, read the appropriate sections before making your selection.

☒ Life sciences ☐ Behavioural & social sciences ☐ Ecological, evolutionary & environmental sciences

For a reference copy of the document with all sections, see nature.com/documents/nr-reporting-summary-flat.pdf

Life sciences study design

All studies must disclose on these points even when the disclosure is negative.

Sample size	At least n = 3 were used for each experimental group, and the exact number of animals for every experiment is indicated in the corresponding figure panels or legends. Sample sizes were chosen based on standard practice in the field and were sufficient to obtain consistent and reproducible results across independent experiments.
Data exclusions	No data were excluded from the analyses.
Replication	Experiments were independently repeated, and replicate numbers are specified in each figure legend. All replication information is fully reported in the manuscript and Supplementary Information.
Randomization	Mice were genotyped prior to experiments and then allocated to experimental groups by simple random assignment. Age- and sex-matched animals were used across groups to minimize potential confounding factors.
Blinding	After genotyping of mice, Investigator was blinded to group allocation and data collection in in vivo experiments. Experiment and further data analysis were performed by person blinded to which animal was being assigned

Reporting for specific materials, systems and methods

We require information from authors about some types of materials, experimental systems and methods used in many studies. Here, indicate whether each material, system or method listed is relevant to your study. If you are not sure if a list item applies to your research, read the appropriate section before selecting a response.

Materials & experimental systems

n/a	Involved in the study	
☐	☒	Antibodies
☐	☒	Eukaryotic cell lines
☒	☐	Palaeontology and archaeology
☐	☒	Animals and other organisms
☒	☐	Clinical data
☒	☐	Dual use research of concern
☒	☐	Plants

Methods

n/a	Involved in the study	
☒	☐	ChIP-seq
☐	☒	Flow cytometry
☒	☐	MRI-based neuroimaging

Antibodies

Antibodies used	Flow cytometry antibodies<br>Lineage Cocktail–APC (BD Biosciences, #558074)<br>Anti-mouse CD45–FITC (BioLegend, #103108)<br>Anti-mouse CD4–PerCP/Cy5.5 (BioLegend, #116012)<br>Anti-mouse CD90.2–BV510 (BioLegend, #140319)<br>Anti-mouse CD127 (IL-7Rα)–PE-Cy7 (BD Biosciences, #560733)<br>RORγt–BV421 (BD Biosciences, #562894)<br><br>Immunofluorescence antibodies<br>Mouse anti-GLP-1 (Novus Biologicals, #NBP2-23558AF488)<br>Recombinant anti-Insulin antibody (Abcam, #EPR17359)<br>Secondary antibody: anti-rabbit IgG (H+L)–Alexa Fluor 647 (Cell Signaling Technology, #4414)<br><br>ChIP antibody<br>Anti–phospho-STAT3 (Tyr705) (Cell Signaling Technology, Danvers, MA, USA; #9145)
Validation	All antibodies used in this study were commercially obtained and validated by the respective manufacturers for the indicated applications. Flow cytometry antibodies from BD Biosciences, BioLegend, and Invitrogen are validated for mouse reactivity and are widely used in the field for the identification of lymphoid and innate lymphoid cell subsets. Immunofluorescence antibodies (anti-GLP-1, Novus; anti-insulin, Abcam) were validated by the vendors for murine IF staining, and produced specific signals restricted to known hormone-producing cells, consistent with established biological patterns. The anti–phospho-STAT3 (Tyr705) antibody (Cell Signaling Technology, #9145) is validated by the manufacturer for ChIP assays and demonstrated expected enrichment at STAT3-responsive genomic regions in our samples. All antibodies were used at vendor-recommended concentrations, which were further confirmed empirically in our laboratory.

Eukaryotic cell lines

Policy information about cell lines and Sex and Gender in Research

Cell line source(s)	STC-1, ATCC
Authentication	Cell lines were authenticated based on morphology, growth characteristics, and expression of known lineage-specific markers, and were consistent with published descriptions of each line.
Mycoplasma contamination	All cell lines used in this study were routinely tested for mycoplasma contamination and were confirmed to be mycoplasma-free using PCR-based detection assays.
Commonly misidentified lines<br>(See ICLAC register)	None of the cell lines used are listed as misidentified in the ICLAC register.

Animals and other research organisms

Policy information about studies involving animals; ARRIVE guidelines recommended for reporting animal research, and Sex and Gender in Research

Laboratory animals	All experiments were performed using laboratory mice, including C57BL/6J wild-type controls, Il22ra1^fl/fl mice crossed with Vil1-Cre or Gcg-Cre, and Gcg-Cre × ROSA-YFP reporter mice. Mice were at least 8 weeks old at the start of experiments and were maintained under specific pathogen–free (SPF) conditions in individually ventilated cages at 20–22 °C, 40–60% humidity, and a 12-h light/dark cycle. All procedures were approved by the Institutional Animal Care and Use Committee of Kangwon National University and conducted in accordance with institutional and national ethical guidelines.
Wild animals	No wild animals were used in this study.
Reporting on sex	Male mice aged at least 8 weeks were used for all in vivo experiments. A single sex was selected to minimize variability in metabolic and hormonal phenotypes and to maintain consistency across experimental groups.
Field-collected samples	No field-collected or environmental samples were used. All biological materials were obtained from laboratory mice or commercially sourced cell lines.
Ethics oversight	All animal experiments were reviewed and approved by the Institutional Animal Care and Use Committee of Kangwon National University (IACUC admission number KW-220808-1, KW-210826-2) and were performed in accordance with institutional and national ethical guidelines for the care and use of laboratory animals.

Note that full information on the approval of the study protocol must also be provided in the manuscript.

Plants

Seed stocks

Not applicable; this study did not use any plant materials or seed stocks.

Novel plant genotypes

Not applicable; no plant genotypes or plant-related genetic modifications were generated in this study.

Authentication

Not applicable; no plant materials were used.

Flow Cytometry

Plots

Confirm that:

☒ The axis labels state the marker and fluorochrome used (e.g. CD4-FITC).

☒ The axis scales are clearly visible. Include numbers along axes only for bottom left plot of group (a 'group' is an analysis of identical markers).

☒ All plots are contour plots with outliers or pseudocolor plots.

☒ A numerical value for number of cells or percentage (with statistics) is provided.

Methodology

Sample preparation

Lamina propria lymphocytes were isolated from mouse small intestines using enzymatic digestion and Percoll gradient separation. Single-cell suspensions were prepared in FACS buffer (PBS with 2% FBS) and filtered through 70-µm strainers prior to staining.

Instrument

Flow cytometry data were acquired using a BD FACS Verse flow cytometer (BD Biosciences).

Software

Data were analyzed using FlowJo software (v10.10.0; BD Biosciences). Compensation was performed using single-stained controls.

Cell population abundance

Immune cell subsets were quantified as a percentage of total live CD45+ cells. Live/Dead dye was used to exclude non-viable cells.

Gating strategy

Cells were gated on FSC/SSC to exclude debris and on FSC-A/FSC-H to remove doublets. Live $CD45^+$ cells were selected, and immune subsets were identified using established Lineage, CD90.2, IL-7R, RORγt, and CD4 markers. Positive and negative boundaries were defined using appropriate controls.

☒ Tick this box to confirm that a figure exemplifying the gating strategy is provided in the Supplementary Information.
